# Mitigating the effects of climate change on the nests of sea turtles with artificial irrigation

**DOI:** 10.1111/cobi.14044

**Published:** 2023-01-22

**Authors:** Christopher R. Gatto, Sean A. Williamson, Richard D. Reina

**Affiliations:** ^1^ School of Biological Sciences Monash University Clayton Victoria Australia; ^2^ School of Biological Sciences Florida Atlantic University Boca Raton Florida USA

**Keywords:** climate change, cooling conservation, embryonic development, irrigation, nest, oviparous, watering, cambio climático, conservación, desarrollo embrionario, enfriamiento, irrigación, nido, ovíparo, riego, 巢穴, 灌水, 气候变化, 卵生, 胚胎发育, 保护, 浇水, 冷却

## Abstract

For sea turtles, like many oviparous species, increasing temperatures during development threaten to increase embryonic mortality, alter offspring quality, and potentially create suboptimal primary sex ratios. Various methods are being implemented to mitigate the effects of climate change on reproductive success, but these methods, such as breeding programs, translocations, and shading, are often invasive and expensive. Irrigation is an alternative strategy for cooling nests that, depending on location, can be implemented relatively quickly and cheaply. However, multiple factors, including ambient conditions, nest substrate, and species characteristics, can influence irrigation success. Additionally, irrigation can vary in duration, frequency, and the volume of water applied to nests, which influences the cooling achieved and embryonic survival. Thus, it is critical to understand how to maximize cooling and manage risks before implementing irrigation as a nest‐cooling strategy. We reviewed the literature on nest irrigation to examine whether artificial irrigation is feasible as a population management tool. Key factors that affected cooling were the volume of water applied and the frequency of applications. Embryonic responses varied with species, ambient conditions, and the timing of irrigation during development. Nest inundation was the key risk to a successful irrigation regime. Future irrigation regimes must identify clear targets, either primary or adult sex ratios, that maximize population viability. Monitoring population responses and adjusting the irrigation regime in response to population characteristics will be critical. Most studies reported on the manipulation of only one or two variables, further research is required to understand how altering multiple factors in an irrigation regime influences the cooling achieved and embryonic responses.

## Resumen

Como sucede con muchas especies ovíparas, el incremento en las temperaturas durante el desarrollo embrionario puede aumentar la mortalidad embrionaria, alterar la calidad de la descendencia y tiene el potencial de crear proporciones sexuales primarias poco óptimas en las tortugas marinas. Se están implementando varios métodos para mitigar los efectos del cambio climático sobre el éxito reproductivo, aunque dichos métodos (p. ej.: los programas de reproducción, reubicaciones y sombreado) suelen ser invasivos y costosos. La irrigación es una estrategia alternativa para el enfriamiento de los nidos, la cual, según la ubicación, puede implementarse de manera rápida y económica. Sin embargo, factores como las condiciones ambientales, el sustrato de anidación y las características de la especie pueden influir sobre el éxito de la irrigación. Además, la duración, frecuencia y volumen del agua aplicada a los nidos durante la irrigación puede variar, lo que influye sobre el enfriamiento y la supervivencia embrionaria. Por todo esto, es importante entender cómo maximizar el enfriamiento y gestionar los riesgos antes de implementar la irrigación como estrategia de enfriamiento de nidos. Revisamos la literatura sobre la irrigación de nidos para analizar si la irrigación artificial es una herramienta viable de manejo poblacional. Los factores clave que afectaron el enfriamiento fueron el volumen aplicado de agua y la frecuencia de las aplicaciones. Las respuestas embrionarias variaron según la especie, condiciones ambientales y el momento de irrigación durante el desarrollo. El principal riesgo para un régimen exitoso de irrigación fue la inundación del nido. Los próximos regímenes de irrigación deben identificar objetivos claros, ya sean las proporciones sexuales adultas o primarias, que maximicen la viabilidad poblacional. Para esto, serán muy importantes el monitoreo de las respuestas poblacionales y el ajuste del régimen de irrigación en respuesta a las características de la población. La mayoría de los estudios reportaron la manipulación de una o dos variables, por lo que se requiere de mayores estudios para entender cómo la alteración de varios factores en el régimen de irrigación influye sobre el enfriamiento obtenido y las respuestas embrionarias.

## 【摘要】

对于海龟来说, 就像许多卵生物种一样, 发育过程中温度升高可能增加胚胎死亡率, 改变后代质量, 并可能导致非最优的原始性别比例。目前人们正在实施各种方法以减轻气候变化对繁殖成功的影响, 如繁殖计划、迁地、遮阳, 但这些方法往往是侵入性的, 而且费用高昂。灌水是一种冷却巢穴的替代策略, 可以根据不同的地点, 相对快速和低成本地开展行动。然而, 有多种因素影响着灌水的成功, 包括环境条件、巢穴基质和物种特性。此外, 灌水的持续时间、频率和用水量也存在变化, 影响着冷却效果和胚胎存活率。因此, 在利用灌水进行巢穴冷却之前, 了解最大限度实现冷却的方法和可能的管理风险是至关重要的。本研究回顾了有关巢穴灌水的文献, 分析了人工灌水作为种群管理工具的可行性。我们发现, 影响冷却效果的关键因素是灌水的量及频率。胚胎的响应随物种、环境条件和发育过程中的灌水时间而有所不同。巢穴被淹没是成功灌水的关键风险。未来的灌水方式必须确定明确的目标, 无论是针对原始性别比例还是成年性别比例, 都应最大限度地提高种群生存力。此外, 监测种群响应并根据种群特征调整灌水方式也十分关键。大多数研究只报告了对1或2个变量进行操作, 需要进一步研究以了解改变灌水方式中的多个因素将如何影响冷却效果及胚胎的响应。**【翻译: 胡怡思; 审校: 聂永刚】**

## INTRODUCTION

Climate change threatens to alter species’ distributions, life histories, and reproductive success (Morán‐Ordóñez et al., 2018; Telemeco et al., 2013). Of particular concern is the effect that climate change will have on reproductive success because warmer temperatures can alter developmental rates, developmental success, and offspring phenotypes, leading to reduced offspring production and survival (Hawkes et al., 2014). Some oviparous species, including sea turtles, are particularly vulnerable to increasing developmental temperatures because, unlike viviparous species, they are unable to physiologically mitigate elevated temperatures by controlling their own internal temperature (Beltrán et al., 2021). Although some oviparous species behaviorally modify the incubation environment (Balshine, 2012), sea turtles provide no parental care, and thus, ambient conditions directly determine the incubation environment.

As global temperatures rise, the reproductive success of sea turtles is expected to be negatively affected in three main ways. First, incubation temperatures are increasingly reaching lethal levels, resulting in elevated embryonic mortality (Santidrián Tomillo et al., 2015). Second, higher temperatures are likely to alter offspring phenotypes, which may reduce survival (Wood et al., 2014). Third, for species with temperature‐dependent sex determination (TSD), elevated developmental temperatures may result in extremely biased primary sex ratios (PSRs) and could eventually result in a lack of reproductive adult males (Hays et al., 2017). Thus, elevated temperatures during development have multiple consequences for hatchling production and survival, and population viability.

To counteract these effects, conservationists globally are seeking strategies to mitigate rising incubation temperatures so as to ensure the continued production of viable offspring (Wood et al., 2014). Common interventions include breeding programs, nest shading, and translocation (Fuentes et al., 2012; Hogg, 2013). These strategies can be expensive and invasive. Shade structures potentially interfere with other species when built over large areas, translocation increases the risk of movement‐induced embryonic mortality, and breeding programs alter hatchling behavior and survival (Booth et al., 2020b; Limpus et al., 1979; Williamson et al., 2017). An alternative strategy for cooling clutches of eggs is to irrigate nests. Irrigation can be conducted in situ (eggs are undisturbed), is minimally invasive, and, depending on the method of application, is relatively inexpensive. However, the efficacy of irrigation depends on multiple factors and, if applied inappropriately, could negatively affect embryonic development (Limpus et al., 2020). For example, overirrigation causing excessive nest water content (NWC) can restrict oxygen availability to embryos, resulting in elevated mortality (Cedillo‐Leal et al., 2017). Thus, the use of irrigation to cool nests requires careful planning and should be implemented only when likely to succeed and when alternate methods are not feasible.

We searched the literature for studies in which sand and sea turtle nests were irrigated to determine the method's efficacy and provide a framework for the development of future irrigation projects. We also present an example of how this framework can be applied to plan and implement a regime of nest irrigation on a sea turtle nesting beach. Where possible we drew on studies in other taxa so that our framework could be applied to multiple taxa, particularly those that bury or cover their eggs, such as crocodiles, tuataras, and some squamates and some birds. We focused on PSR and embryonic mortality because they are predicted to have the greatest consequences for population viability and have, therefore, been the focus of most studies (e.g., Santidrián Tomillo et al., 2015). Finally, we devised recommendations for addressing potential sources of variation in irrigation efficacy and identified species that may benefit from irrigation.

## METHODS

To find all relevant information on nest irrigation, we searched the literature for all studies that applied water to nests during incubation and recorded the response of the incubation environment. We also included studies that recorded the response of nests and offspring to rainfall, tidal over wash, flooding, and other water sources. We searched Web of Science, Google Scholar, and article reference lists for study titles that contained the words *nest*, *eggs* OR *clutch* combined with *irrigation*, *watering*, *sprinkling*, *rainfall* OR *flooding*. We also requested data from other researchers via the CTURTLE list server (https://accstr.ufl.edu/resources/cturtle/). Studies that reported only the response of offspring traits but did not report the response of the incubation environment were excluded from the analyses. Seven and three studies reported the response of the nest environment to irrigation and rainfall, respectively. No studies reported the response of the nest environment to flooding.

## DYNAMICS OF NEST IRRIGATION

Unless water can be applied directly to eggs via pipes, irrigation generally involves watering the substrate surface so water percolates through the substrate to the nest (Matthews et al., 2021; Smith et al., 2021). As the water percolates, its temperature equilibrates to that of the surrounding substrate. Depending on the substrate temperature, this can affect the water's cooling potential (Lolavar & Wyneken, 2015). If the water is cooler than the nest, it directly absorbs heat from the eggs and substrate as it percolates through the sand via bulk flow (Sakura, 1984). In contrast, water that is warmer will heat the nest. Higher NWC results in greater evaporative cooling potential (Ackerman et al., 1985). Generally, temperature and moisture are more stable at greater depths (Ackerman & Lott, 2004), and substrate at the surface tends to dry and heat first; the “dry front” and temperature reach greater depth over time (Swiggs et al., 2018). Thus, a substrate's surface temperature and moisture can differ significantly from that at nest depth. Substrate characteristics (e.g., grain size and thermal conductivity) affect the dynamics of water, gases, and temperature in and around the nest (Booth et al., 2022; Stewart et al., 2019). Sand, soil, and leaf litter, for example, have different thermal properties and interact with moisture changes differently (Hillel, 2003). Generally, substrates with smaller grain sizes and pore spaces can hold larger volumes of water but limit the movement of water and gases, which affects the amount of cooling from irrigation.

## EFFECTS OF NWC

Irrigation is designed to cool nests to increase offspring production, produce offspring with particular traits (e.g., sex), or both. However, irrigation decreases the temperature and usually results in higher NWC (Erb et al., 2018; Hill et al., 2015), and NWC can influence other environmental variables, including PO_2_ and salinity (Booth, 1998; Foley et al., 2006). Gatto and Reina (2022) reviewed the effect that changes in NWC have on other environmental variables. Generally, elevated NWC results in lower temperature and oxygen availability (Ackerman, 1997). Freshwater irrigation flushes salts from the substrate, whereas seawater can deposit salts (Foley et al., 2006). Substrate type can alter these relationships and embryonic responses (Hillel, 2003). We did not focus on the direct effects of moisture on embryos because the response of nests and embryos to altered NWC depends on multiple factors, including species, substrate, and ambient conditions. However, NWC may directly influence sex determination in sea turtles (Lolavar & Wyneken, 2017, 2020). Gatto and Reina (2022) extensively reviewed the effects of moisture on reproductive success and offspring traits in nonsquamate reptiles. There are currently no reviews on birds or insects, but moisture affects offspring phenotypes in both (Klimstra et al., 2009; Norhisham et al., 2015).

## ARTIFICIAL NEST IRRIGATION

Artificial irrigation affects the incubation environment, development, and offspring traits in sea turtles (studies listed in Table 1). There are few studies of this for other taxa. Studies on the effects of shading and cooling from rainfall are more common and include more species, mainly reptiles. Studies are limited to birds, but artificial rainfall reduces the insulation capacity of songbird nests, resulting in cooler nest temperatures (Deeming & Campion, 2018). As temperatures become warmer, bird parents are likely to spend less time on eggs (Coe et al., 2015), providing greater opportunities for irrigation. However, due to the paucity of studies on other taxa, we focused on studies of sea turtles. To account for differences in the area around individual nests that was irrigated, we report irrigation volumes in millimeters of rainfall equivalent applied to each plot. This allowed for direct comparisons with rainfall effects. We refer to the application of water to incubating eggs as *watering nests* and to the application of water to substrate only (i.e., no eggs present) as *watering substrate*.

**TABLE 1:** Summary of the turtle irrigation regimes implemented in studies included in a review of whether artificial irrigation is feasible as a population management tool and the response of the nest environment and offspring traits to irrigation.^a^

					Timing of irrigation		Irrigation regime						
Study type	Study	Irrigation target, species (irrigation depth, cm)	Sun and shade exposure	Water source	time of day (water temp.,°C)	time during development	frequency	duration (days)	volume (mm/day)	Nest water content ^b^	Temperature increase (+)_ or decrease (−), °C	Sex ratio, % females	Hatching success (%)
Irrigation	Smith et al. (2021)	sand and eggs, green (70 cm)	sun and shade	freshwater and seawater	dawn (24.2–25.1)	day 18, before thermosensitive period	both one‐off and daily	1, 4, and 7	20, 50, 100, and 200	not reported	+0.1 to 0.5 to −1.3 (shade treatment ∼0.3° cooler than sun exposed)	100 (logistic model)	74.9–92.4
	Hill et al. (2015)	sand (45 and 75 cm)	sun and shade	freshwater	afternoon	no eggs	daily	31	3.2, 10.4, and 23.3	1.3–8.5% w/w	−0.6 to −4 (shade treatment 2.2 cooler than sun‐exposed)	no eggs	no eggs
	Jourdan and Fuentes (2015)	sand in plastic containers (50 cm)	sun and shade	freshwater	noon (34) and night (25)	no eggs	daily	12	50 (noon) and 100 (night)	not reported	+0.83 to −2.23 (shade ∼0.68 cooler than sun on nonrainy days and ∼0.96 warmer on rainy days)	no eggs	no eggs
	Lolavar and Wyneken (2021)	eggs, loggerhead (60 cm)	sun	freshwater	morning	entirety of dev.	daily	entirety of dev.	40, 80, and 140	∼5% to 8% v/v	−0.2 to −1.5	71–100 (laparoscopic examination)	45–90
	Erb et al. (2018)	eggs, loggerhead (60 cm)	sun	freshwater	morning	entirety of dev.	daily	entirety of dev.	40	5.3% (control) and 6.8% v/v (water treatment)	−0.16	not reported	not reported
	Matthews et al. (2021)	eggs, loggerhead (70 cm)	shade	freshwater	not reported	entirety of dev.	as required	entirety of dev.	as required, 4–9 mm	4.89% (low moisture treatment) and 7.89% v/v (high moisture treatment)	−0.83	∼70 and ∼90 (logistic model)	94.4 and 95.6
	Bodensteiner et al. (2015)	eggs, painted turtle (7 cm)	not reported	freshwater	not reported	majority of dev. (not the last few days)	twice weekly	majority of dev. (not the last few days)	30	11–32% v/v	−0.1 to −0.9	39 and 44 (generalized linear mixed model with binomial distribution and logit link function)	52–82
Rainfall ^c^	Laloë et al. (2021)	eggs, green (70 cm)	sun	rain	not reported	not reported	once	4	129.8 (519 total)	not reported	−2.3 and −4.3	not reported	not reported
	Staines et al. (2020)	eggs, hawksbill (50 cm) and green (65 cm)	sun and shade	rain	not reported	not reported	one‐off	2	62.5 (125 total)	not reported	−3.1 to −4.1 (prerainfall, shade ∼0.9 and ∼1.3 cooler than sun‐exposed nests for hawksbill and green turtles, respectively)	not reported	not reported
	Lolavar and Wyneken (2015)	sand and eggs (30 and 45 cm)	sun	rain	not reported	some during thermosensitive period	one‐off	not reported	60–143	not reported	as high as −7	0–100 (laparoscopic examination)	4–99
Inundation	Limpus et al. (2020)	eggs, loggerhead (50 cm)	not reported	freshwater and seawater	not reported	0, 1, 2, 4, 6, and 7 weeks	one‐off	0, 1, 2, 3, 6, 24, and 48 h of inundation	complete inundation	not reported	not reported	not reported	0–100%
	Patino‐Martinez et al. (2014)	eggs, leatherback (no depth)	not reported	freshwater	not reported	start of dev.	one‐off	moisture set and then not adjusted for the rest of dev.	volume added to create specific water content	1%, 2%, 6%, 10%, & 12% w/w	not reported	not reported	0–65%
	Pike et al. (2015)	eggs, green (no depth)	not reported	saltwater, 28 ppt	not reported	0%, 33%, 50%, and 66% of dev.	one‐off	1, 3, and 6 h of inundation	complete inundation	not reported	not reported	not reported	30–70%
	Foley et al. (2006)	eggs, loggerhead (15, 30, and 45 cm)	not reported	saltwater, water salinity: 26.9 ppt, nest: 16.8 ppt (15.7–67.9)	not reported	not reported	no inundations, one‐off and multiple	not reported	no inundation, part inundation, and full inundation	1–19% w/w	not reported	not reported	20.5–84.4%
	Ware and Fuentes (2018)	eggs, loggerhead (no depth)	not reported	seawater	not reported	not reported	relocated clutches: 1.5 inundation and 0.7 wash overs in situ: 1.9 inundations and 1 wash over	not reported	wash over and complete inundation	2.66% and 2.9% w/w	relocated clutches were 0.4°C cooler	no sex ratios; mean temp. of the middle third of dev.: 29.6 and 29.7	65.8% and 66.3%

^a^ Appendix S1 details how likely it is that each factor of the irrigation regime (e.g., water source) influences nest environment (e.g., salinity) and reproductive success and hatchling traits (e.g., hatching success).

^b^ Key: % v/v, volume of water in a sample divided by the volume of the substrate in a sample; % w/w, weight of water in a sample divided by the weight of the substrate in a sample.

^c^ For studies presenting the effects of rainfall, included are data only for rainfall events for which the amount of cooling was reported for a specific volume of rain in a single event, rather than for the total amount of rain over an entire nesting season.

### Irrigation method

**FIGURE 1:**
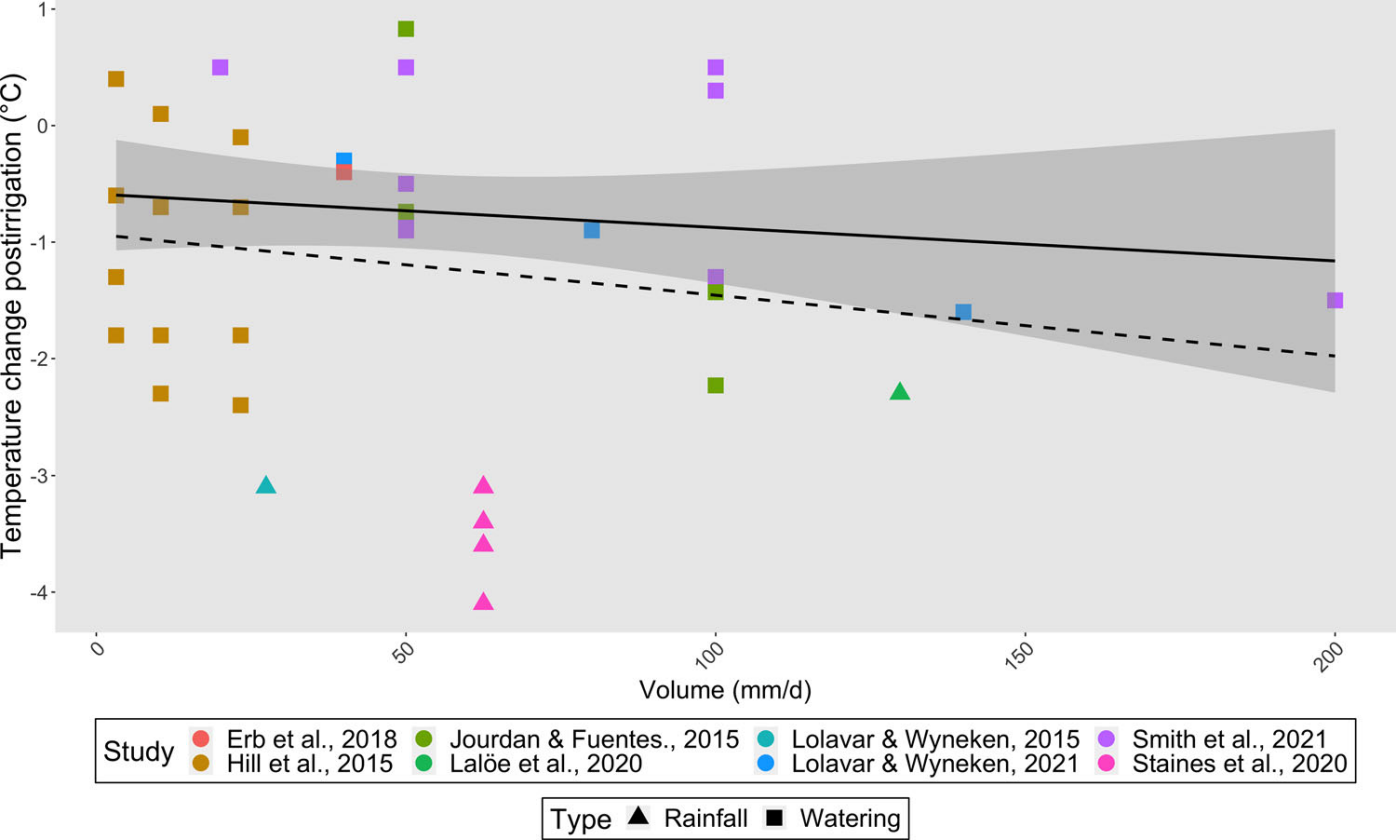
Relationship between the volume of water used to irrigate a turtle nest and the amount of cooling achieved (data from the irrigation of sand only and of clutches of eggs; irrigation volume in mm/day rainfall equivalent; solid line, best fit for artificially irrigated nests [*n* = 34]; dashed line, best fit includes nests that were irrigated by rainfall [*n* = 8, total nests 42]; shaded area, 95% confidence intervals of the line of best fit for artificially irrigated nests only). Lines of best fit were generated by the stat_smooth function in the R package ggplot2 (Wickham, 2016). The effect of water volume on the amount of cooling was determined using linear mixed‐effects models with volume as the fixed effect and type (watering or rainfall) and study as random effects. Volume had a significant effect on the amount of cooling (*t*_36.4_ = −2.05, *p* = 0.048), and the type of irrigation and study explaining 74.5% and 6.8% of the data, respectively. Models were run in lme4 package in R (Bates et al., 2015).

Water is typically applied from the surface with a watering can (Bodensteiner et al., 2015) or sprinkler (Erb et al., 2018). We found no studies that reported underground irrigation of nests. Underground irrigation may be limited to species whose nest location is predictable and underground pipes will not be damaged by nesting females or affect the ability of females to dig (Hays & Speakman, 1993). Surface irrigation did not always result in reduced temperatures in green turtle (*Chelonia mydas*) nests at a depth of 70 cm, likely because the warmer surface substrate heated the water to temperatures above those at nest depth as it percolated downwards (Smith et al., 2021). Generally, rainfall provided greater cooling effects than artificial irrigation for an equivalent quantity of water (Figure 1 & Table 1). Artificial irrigation generally decreased temperatures to <2°C, whereas rainfall consistently cooled nests by ≥2°C. This difference may result from shade from overcast skies, rain being cooler than water sitting in pipes or buckets, or rain watering entire beaches, increasing evaporative cooling potential and reducing water loss from irrigated nests to surrounding drier sand.

### Effect of shade and nest depth

Irrigation and rainfall had less effect on substrate temperature as depth increased. Under three watering regimes, sun‐exposed sand (hereafter exposed sand) at 45 cm depth decreased in temperature by 2.2°C on average, compared with a 0.7°C decrease at 75 cm (Hill et al., 2015). Surface irrigation cooled shaded sand less effectively than exposed sand. For example, exposed sand at 45 cm depth generally cooled 0.5°C more than shaded sand when irrigated with different volumes of water (Hill et al., 2015). Differences between exposed and shaded nests may result from the already lower temperatures of shaded nests, limiting their ability to be further cooled, or from evaporative cooling, which is low in shaded nests. Shade structures may limit rainfall's cooling benefits because they keep nests dry (Staines et al., 2020). Shading typically reduces nest temperatures by up to 2°C (Reboul et al., 2021; Wood et al., 2014) and is generally more effective at reducing temperatures than irrigation (Table 1). However, compared with shading, irrigation does not require substantial infrastructure and can simultaneously cool nests and increase NWC, which may be beneficial on dry beaches.

### Water source

Regardless of the method used to irrigate nests, investigators typically used freshwater, normally from taps or rainfall (Table 1). Only Smith et al. (2021) used both seawater and freshwater and found no difference in cooling. Hatching success was generally higher in nests irrigated with freshwater than seawater, likely because deposited salts interfere with physiological processes (D. Booth. 2021 Presentation to the Raine Island Scientific Advisory Group; Smith et al., 2021). However, in Smith et al. (2021), seawater (71.2% hatching success) and freshwater (83.8%) treatments did not differ statistically from each other or compared to exposed, control nests (63.5%). Early‐stage (<20% developed) and late‐stage (>80%) embryos are more sensitive to seawater inundation when eggs are submerged in water, than when embryos are at intermediate developmental stages (Limpus et al., 2020). Both freshwater and seawater inundation resulted in almost complete mortality in early‐stage embryos (<12%). Embryos at intermediate stages do not differ in their sensitivity to seawater and freshwater (Limpus et al., 2020). Late‐stage embryos were not inundated with freshwater, but the higher oxygen demands of late‐stage embryos suggest that freshwater inundation would increase mortality. Inundation is an extreme condition usually caused by wave overwash or rising water table. It is unlikely that well‐managed irrigation will inundate nests, but monitoring NWC is critical. In locations with high substrate microbial loads, seawater irrigation may improve hatching success by reducing microbial abundance (Bézy et al., 2015).

### Irrigation volume

Determining the relationship between the volume of water applied and its effect on nest temperature is complicated by the wide range of regimes used (Table 1). Greater volumes applied to sand and nests generally resulted in larger cooling effects in and among studies (Figure 1), but the amount of additional cooling achieved varied (Hill et al., 2015; Smith et al., 2021). The effect of large volumes of water applied once or intermittently on PSRs was less consistent than daily irrigation because their effect depended on the timing of irrigation relative to the thermosensitive period (TSP) (Porter et al., 2021).

Irrigation negatively affects hatching success if nest moisture becomes too high or inundation occurs (Limpus et al., 2020; Lolavar & Wyneken, 2021). Thus, understanding how irrigation influences substrate water content and the capacity of clutches to tolerate elevated NWC is vital. Generally, irrigating with higher volumes resulted in higher sand moisture (Smith et al., 2021). However, this effect was not consistent (Lolavar & Wyneken, 2021), and water accumulation within the substrate may be influenced by factors, such as substrate type, evaporative rates, and vegetation. Deeper nests generally increased in moisture more than shallower nests during irrigation (Hill et al., 2015). As water is applied to the surface, it percolates down through the substrate, where it eventually reaches equilibrium with the capillary forces between sand grains (Hill et al., 2015). Thus, the surface substrate initially increases in water content before drying as the water drains down through the substrate and evaporates at the surface. In comparison, deeper nests experience lower evaporative rates and are closer to the water table, so water equilibrates at higher concentrations.

### Timing of irrigation

The temperature of the water applied and the time of application may be as important for cooling as volume. Water is most often reported as being applied in the morning, but irrigation also occurred at noon, late afternoon, and night (Table 1). Morning irrigation appears to achieve the most cooling, most likely because substrate surface temperatures are the coolest. If water is applied when substrate surface temperatures are hotter than at nest depth, the applied water can heat to temperatures above those at nest depth, resulting in an increase in nest temperature (Smith et al., 2021). Irrigating in the morning, when surface temperatures are coolest, minimizes this risk. Additionally, in remote locations or where large volumes are required, water will need to be stored, most likely in tanks. Water stored in direct sunlight or hot temperatures will inevitably warm, decreasing its cooling potential. Strategies to keep stored water cool will be important, and possibilities include burying water tanks underground, insulated tanks, and cooling systems.

**FIGURE 2:**
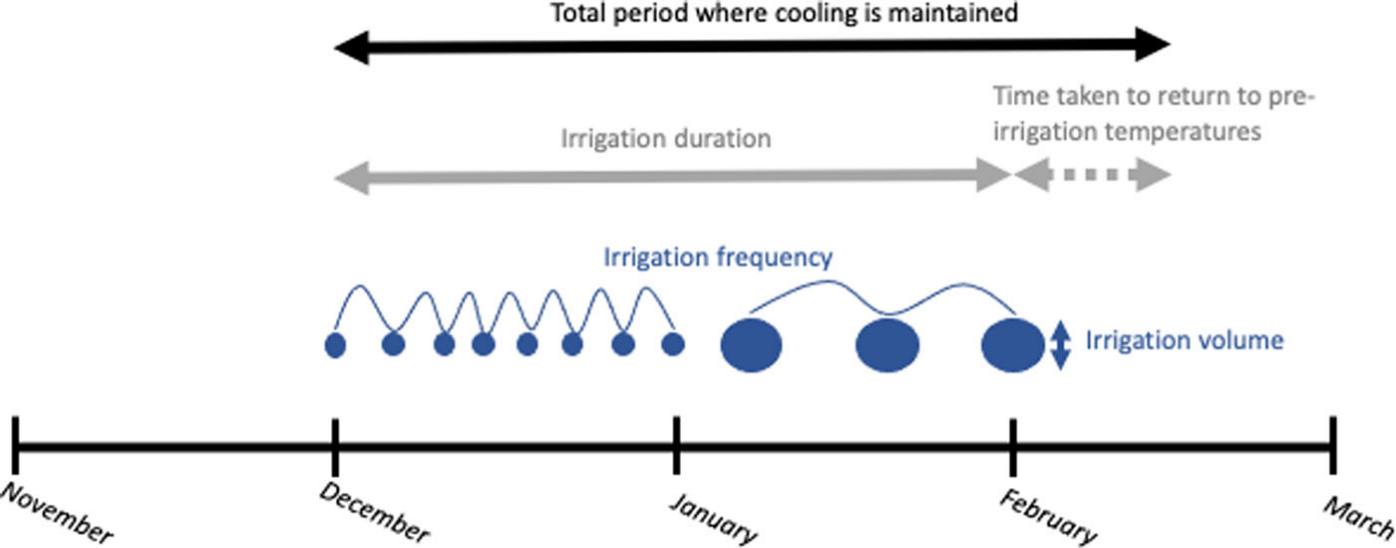
Visual representation of common descriptions of turtle nest irrigation we used (circles, applications of water at different times in the nesting season; circle size, proportional irrigation volume). Irrigation duration is the length of time nests were regularly irrigated (nests were irrigated from December to February). Irrigation duration can also be expressed in terms of development (e.g., nests were irrigated throughout development or just during the thermosensitive period). Irrigation frequency is how often nests are irrigated throughout the irrigation period (e.g., daily and weekly).

Frequency, duration, and when (timing) irrigation occurs are all part of irrigation timing (Figure 2). Frequency has been either a single application, daily irrigation, or targeted irrigation, where irrigation frequency or volume is varied to maintain predetermined incubation conditions (Matthews et al., 2021). All three frequencies produced cooling effects (Table 1). However, it typically takes 4–10 days after irrigation stops for nest and substrate temperatures to return to preirrigation levels (Hill et al., 2015; Laloë et al., 2021; Lolavar & Wyneken, 2015). Effects can last for 20 days in some cases, most likely depending on sand characteristics and ambient climatic conditions (Staines et al., 2020). Thus, the benefit of daily or frequent irrigation compared with single applications is that lower, more stable temperatures can be maintained. Single applications cool nests for 4–10 days, whereas daily irrigation maintains cooling for the duration of irrigation plus 4–10 days postirrigation (Figure 2).

Regular irrigation reduces long‐term temperature fluctuations by reducing nest temperatures and limiting exposure to high temperatures. However, short‐term, daily irrigation can increase the size of temperature fluctuations by rapidly reducing nest temperatures each day (Jourdan & Fuentes, 2015). This increase in short‐term temperature variation can have considerable consequences for PSRs. For example, in sea turtles, greater thermal variation results in more females being produced than expected based on mean nest temperatures (Georges et al., 1994), unless the temperature always remains outside the transitional range of temperatures (TRT) (range of temperatures under which PSR changes from 100% males to 100% females). Sex determination depends on the proportion of embryonic development that occurs at each temperature, and development rates are faster at higher temperatures. Thus, if the same amount of time is spent at a warm and a cool temperature, a greater proportion of development will occur at the warm temperature (Georges et al., 1994). Therefore, larger fluctuations in incubation temperature, above and below the mean temperature, will result in a greater proportion of development occurring at the temperatures above the mean temperature and more female hatchlings being produced than under stable temperatures (Georges et al., 1994). This may only be problematic for irrigation regimes that shift temperatures only slightly downward and in populations that already produce both sexes. In locations where PSRs are 100% females, the effect of temperature fluctuations is moot, and if irrigation significantly decreases nest temperature, then male production will increase, although the effect will not be as large as expected. Greater diel thermal variation can result in reduced embryonic survival if fluctuations exceed tolerable limits (Howard et al., 2014).

Irrigation durations ranged from single applications to the entirety of incubation (Table 1). Duration does not appear to influence how long cooling effects last once irrigation ceases, but regular irrigation ensures that reductions in temperatures are maintained until irrigation ends (Figure 2). Thus, regular irrigation throughout incubation ensures that cooling effects are maintained for the entirety of development, whereas shorter irrigation durations may need to target the TSP to change PSR. For example, Smith et al. (2021) found that a single application of 100 mm of water on day 18 of incubation did not alter PSRs in green turtles because temperatures were still too high during the TSP. If nests had been watered daily throughout the TSP, then it is possible that more male hatchlings would have been produced irrespective of when the nests were laid. In natural settings, unless a species exhibits synchronized reproduction, it is unlikely that the developmental stage will be known for all nests, making it difficult to target the TSP for individual clutches. In these situations, ongoing and regular irrigation is required to ensure that cooling is achieved during the TSP in most nests. However, regular irrigation consumes more water and involves irrigating clutches during the early and late stages of development, when embryos are more sensitive to moisture and are outside the TSP (Limpus et al., 2020).

When increased hatching success is the objective rather than PSR, single applications still need to be targeted. The greatest benefit will be gained by irrigating on particularly hot days or at developmental stages when embryos are most at risk to thermal stress, usually early when embryos are more sensitive to temperature and late when nest temperatures are warmer because of metabolic heating (Howard et al., 2014). However, this needs to be balanced with the increased risk of overirrigation (Limpus et al., 2020). During wet nesting seasons or cool portions of the nesting season, irrigation may be required less often or not at all (Lolavar & Wyneken, 2015).

## IMPORTANT FACTORS TO CONSIDER

### Species and population characteristics

Irrigation needs to be tailored for different species and populations because responses to altered NWC during development vary. For example, differences in nest depth can influence temperature and moisture (Hill et al., 2015). In species with TSD, TRT can vary substantially. In sea turtles, TRT varies among populations and is narrow (1–3°C) (Booth et al., 2020b; Porter et al., 2021). Therefore, consistently maintaining mixed PSRs could be difficult. In comparison, the TRT of tuataras is 4—6°C (Nelson et al., 2004), making it easier to maintain mixed PSRs. When the TRT is narrow, it may be more feasible to irrigate nests to achieve a temperature that ensures PSR is 100% male or female for some nests, whereas others are irrigated only to minimize mortality. Likely, some species will be more sensitive to variation in NWC during development (Gatto & Reina, 2020) and will require closer monitoring of moisture and phenotypic responses.

Populations will likely vary in how biased their PSRs and adult sex ratios (ASRs) are, which will alter the cooling required and thus how irrigation should be implemented. Populations with unnaturally, extremely biased PSRs will require greater cooling and thus larger volumes or more frequent irrigation. This will likely increase the risk of overirrigation and produce less‐desirable phenotypes. In populations with extremely biased ASRs, irrigation may need to be implemented longer, either for more of the nesting season or over multiple seasons, to achieve target ASRs. Thus, population PSR and ASR must be estimated before irrigation begins and expected changes in these ratios must be modeled when the changes in incubation temperature are known.

### Determining optimal sex ratio

The ultimate goal of an irrigation regime is usually to alter population ASR or operational sex ratio (OSR) (ratio of actively breeding males to actively breeding females in a population at a given time) by altering PSRs. Thus, irrigation regimes need to focus on ASR and OSR, not solely PSR, so that the PSR selected produces ASRs or OSRs that maximize population viability. For example, sea turtle populations may be naturally female biased because females nest less frequently than males (Hays et al., 2010; Hays et al., 2022), and an ASR of 80 females to 20 males may result in a balanced OSR (Santidrián Tomillo et al., 2021). Thus, attempts to create balanced PSRs may reduce population viability (Santidrián Tomillo et al., 2021) because the OSR will be imbalanced with excess males. Therefore, in this example, irrigation should aim to achieve an ASR of 80% females. This may mean producing a PSR of 80% females over many years. In populations where the ASR is more extreme (e.g., 99% females) (Tanner et al., 2019), it may be necessary to produce PSRs of 100% males until the targeted ASR is achieved. Although OSR is the preferable measure of success for irrigation in sea turtles, ASR can be used if the respective breeding periodicities of males and females in a population are known.

### Trade‐offs among hatching success, PSRs, and other traits

Nest irrigation studies generally focus more on sex determination than subsequent hatching success. However, altering NWC has multiple effects on offspring phenotypes, including size and locomotor performance (Gatto et al., 2021). It is important to monitor and consider the effects of irrigation on multiple traits and not to focus on an individual trait, which can result in negative consequences for ignored traits. For example, if a population is extremely female biased, an irrigation regime is established to produce 100% males. A large amount of cooling is required, so large quantities of water are applied daily to successfully produce 100% males. However, the large volumes decreased hatching success from 75% to 40%. Embryonic development has been negatively affected, resulting in smaller, slower offspring than in previous years. Thus, fewer offspring survive to adulthood than before irrigation. Irrigation regimes that solely monitor PSRs are less likely to identify negative effects, potentially resulting in reduced population viability, despite achieving PSR targets. Regimes that aim to maximize hatching success, without considering PSR, may reduce population viability by producing too many males (Santidrián Tomillo et al., 2021).

### Timing of irrigation during the nesting season

In species with synchronized reproduction, most nests can be irrigated simultaneously because nests are laid at the same time and are at similar stages of development (Hodge et al., 2011). When reproduction occurs over long periods and individual females lay multiple clutches during a nesting season (Reina et al., 2002), it is difficult to ensure that all nests are irrigated at the correct time, but it allows for the duration and timing of irrigation to be altered to achieve desired outcomes at the population level. For example, in species with long nesting seasons, rather than irrigating every nest to produce balanced PSRs, half the nests are irrigated. If irrigated nests are cooled to produce 100% of the less common sex and nonirrigated nests produce 0% of the less common sex, then the population PSR remains balanced. Irrigation should aim to alter ASRs and PSRs at the population level, and not every nest needs to be irrigated. It may be preferable to irrigate only during the nesting peak to manage how many nests are irrigated relative to irrigation effort. Irrigation may target the coolest parts of the nesting season to maximize the production of the less common sex. Alternatively, irrigation may occur only during the hottest months, when nests are most likely to have extremely biased PSRs and high mortality. This targeting of cooler and warmer periods can occur within and among nesting seasons. Irrigating only during cooler years may produce cohorts of offspring that are dominated by the less common sex, which counters years that were dominated by the more common sex (Hays et al., 2022). Irrigating during hotter years minimizes mortality to ensure that the less common sex is produced each nesting season.

It is possible that nests laid at different times are at greater risk of destruction than others. For example, accidental nest destruction by conspecifics is common on high‐density nesting beaches (Bézy et al., 2016); nests are most likely destroyed if laid before or during the nesting peak. The potential for nest destruction may result in irrigation being more effective later in the nesting season when destruction is lower.

## CONSEQUENCES OF OVERIRRIGATING AND NEST INUNDATION

Generally, larger irrigation volumes result in greater cooling. However, the volume applied is limited by each nest's capacity to tolerate elevated NWC during development. Elevated substrate moisture can limit the diffusion of gases leading to hypoxia and hypercapnia (Ackerman, 1997). Elevated moisture could directly interfere with physiological processes, resulting in slowed growth and embryonic mortality (Cedillo‐Leal et al., 2017; Limpus et al., 2020). Thus, it is vital to understand the physiological limits of species being irrigated and to monitor NWC throughout irrigation, especially when irrigating with seawater or water with elevated mineral concentrations or impurities. As water evaporates postirrigation, salts from seawater are deposited into the nest and can accumulate, leading to negative physiological consequences for embryos (Foley et al., 2006). Ensuring nests are not overirrigated must be considered when implementing a regime.

## IMPORTANCE OF MONITORING AND ADJUSTMENT

Monitoring and adjustment are the most important steps in establishing an effective irrigation regime. It is important to determine whether irrigation is necessary by monitoring population traits, including PSR and OSR. This provides a benchmark by which the success of the regime can be measured. Differences among species, ambient conditions, and substrates mean that, even in nearby populations, the same regime can result in different amounts of cooling and responses to irrigation. Thus, once irrigation commences, monitoring nest conditions and embryonic responses is necessary to ensure desired outcomes. Ideally, the variable being manipulated, normally PSR or hatching success, should be monitored directly. This is not always possible, particularly in species that are difficult to identify sex and in highly cryptic species. Direct monitoring of PSRs should become more practical as new sex‐determination techniques are developed (Tezak et al., 2020). Incubation temperature can be used as a proxy for PSR, but PSR can vary among clutches, even in identical incubation environments (Lolavar & Wyneken, 2017). Monitoring moisture and salinity provides data to ensure that embryonic mortality, from inundation or salt concentrations, does not become problematic. Thus, multiple environmental variables should be monitored throughout irrigation. Irrigation volume and frequency can be increased if greater cooling is needed and can be decreased as moisture accumulates in the nest substrate. If salt accumulation becomes problematic, then irrigation frequency, volume, or both should be reduced. Alternatively, seawater could be diluted with freshwater or seawater and freshwater applications could be alternated to ensure that salt does not accumulate.

Once a regime has been established that achieves desired temperatures and PSRs without negatively affecting hatchling production, then fine‐tuning can occur. The aim should be to adjust irrigation to compensate for changes in air temperatures, solar radiation, rain, and other climatic variation. Storm surges may lead to elevated salinity. Irrigation may be unnecessary with high rainfall or low temperatures. In hot, dry years, irrigation can be adjusted when the amount of water needed to achieve the required cooling becomes impractical. In cooler, wetter years, less irrigation is required to achieve targeted temperatures.

## CASE STUDY

**FIGURE 3:**
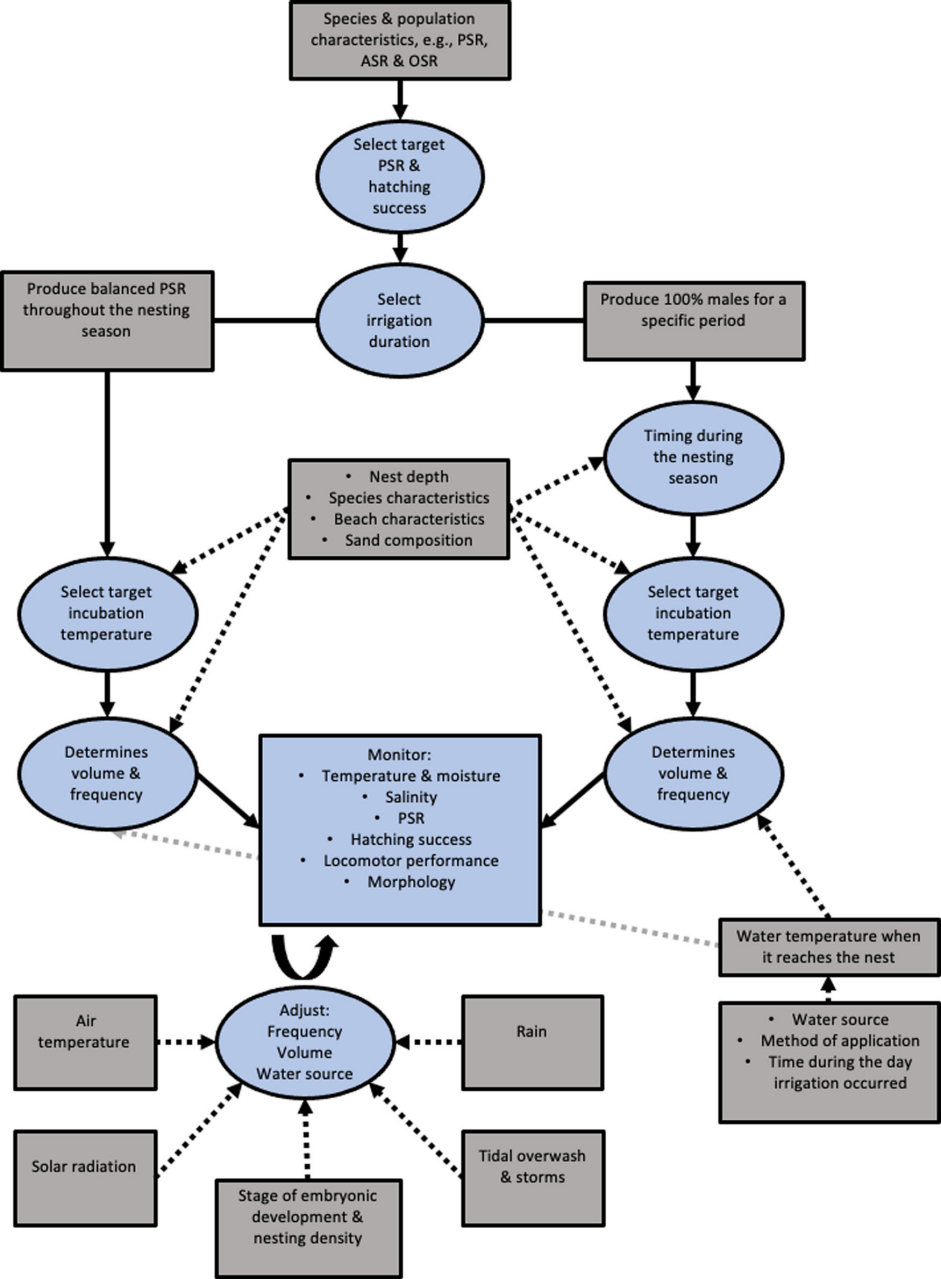
Decision‐making flowchart for establishing and refining an irrigation regime (blue, management and decision‐making stages; gray, factors that influence these decisions). Abbreviations: ASR, adult sex ratio; OSR, operational sex ratio; PSR, primary sex ratio.

To illustrate the planning and execution of an irrigation regime, we applied our irrigation decision‐making framework (Figure 3) and lessons learned from previous studies to green turtle nests on Raine Island in the Great Barrier Reef, Australia. This population is significantly affected by anthropogenic impacts.

### Species and population characteristics

Raine Island hosts the world's largest nesting population of green turtles. Up to 100,000 females can nest there from November to March (Dunstan et al., 2020b). During the 1980s, hatching success was high and consistently above 75% (Limpus et al., 2003). Since then, hatching success has decreased to 33% in some years (Dunstan & Robertson, 2017). Thus, this population is in the early stages of a major decline in reproductive adults (Dunstan & Robertson, 2019), corresponding to declines in hatchling production first reported in the 1990s (Limpus et al., 2003). Population PSR and ASR are estimated to be 99% and 87%, respectively (Jensen et al., 2018). High‐density nesting leads to nest destruction by subsequent nesters or reduced PO_2_ and high incubation temperatures (Dunstan et al., 2020a). Traditional methods to combat elevated temperatures, such as shading, are not feasible because of the lack of trees, large area to be covered, and a large seabird colony. An alternative is an irrigation.

### Selecting PSRs and hatching success

Sea turtle populations are naturally female biased (Hays et al., 2017) because females breed less frequently than males (Hays et al., 2010). Therefore, creating balanced PSRs may result in reduced population viability (Santidrián Tomillo et al., 2021). The narrow TRT in sea turtles makes it difficult to maintain mixed PSRs. It may be more feasible to irrigate nests to achieve a temperature that ensures 100% male production for some nests and to irrigate others only to minimize mortality. This approach would produce 100% males during particular periods, with the aim of producing a PSR of 20% males over a nesting season, which is the PSR predicted to maximize population viability in the leatherback (*Dermochelys coriacea*) population that nests in Playa Grande, Costa Rica (Santidrián Tomillo et al., 2021). Ideally, a population viability analysis (Santidrián Tomillo et al., 2021) would be conducted specifically for the Raine Island population before irrigation. We recommend irrigating nests as needed throughout the nesting season when incubation temperatures reach upper limits.

### Selecting irrigation duration and timing

If aiming to produce 100% males during certain periods, with 100% females probably being produced at all other times, then irrigation should occur when nest temperatures are already lowest to minimize the cooling needed or during peak nesting to maximize male production relative to irrigation effort. We recommend irrigating in October and November. This will result in the target PSR over the entire season because ∼20% of all nests are laid during these months and will reduce late‐stage embryonic mortality in October, when nest temperatures during the last week of development are highest (Table 2). Additional irrigation may be required at the end of the nesting season if nest destruction by conspecifics significantly affects hatchling production. Additional irrigation would be most appropriate in March or April, when temperatures during the TSP are lower, but it may need to occur in February, when nesting numbers are higher, but more cooling is needed to produce more male hatchlings. Nesting peaks in late December to early January; thus, irrigating at this time maximizes the number of nests that can be irrigated in the shortest time. However, nest temperatures during this period are high, so large volumes of water would be required to achieve the necessary cooling. Therefore, we recommend irrigating in October and November when smaller volumes are required.

**TABLE 2:** Number of nesting female turtles, nest conditions, hatching success, and primary sex ratios on Raine Island throughout the nesting season and for two nest irrigation scenarios goals: Produce 100% males in certain months and produce 50% males throughout the nesting season

	October	November	December	January	February	March	April	Comments
Number of females nesting	137	2801	4940	5697	1237	129	10	averages calculated from seasonal technical reports (Dunstan, 2015, 2016; Dunstan & Robertson, 2017, 2018, 2019; Dunstan et al., 2020a; Limpus et al., 2003)
Nests irrigated (%)	1	19	33	38	8	0.8	0.2	
Mean nest temperature (°C) in first week of development	29.5	28.5	31.5	30	30	N/A	30	Booth et al. (2020b)
Mean nest temperature (°C) in thermosensitive period	31	30	30.9	30.8	30.7	N/A	29.7	
Mean nest temperature (°C) last week of development	34.7	32	33	32.5	31.5	N/A	30.5	
Hatching success (%)	65	76	74	57	45	N/A	15	Booth et al. (2020b)
Primary sex ratios (% male)	2.4	3	0.1	0.5	0.5	N/A	8	
Proportion of all offspring that are male	0.02	0.57	0.03	0.19	0.04	N/A	0.02	calculated by multiplying the proportion of nests for each month and the primary sex ratio
Cooling (°C) required to produce 50% males (∼29.1°C)	1.9	0.9	1.7	1.7	1.7	N/A	0.6	
Cooling (°C) required to produce 100% males (∼28°C)	3	2	2.8	2.8	2.8	N/A	1.7	
Mean monthly rainfall (mm/day)	27.8 (0.9)	67.3 (2.24)	206.8 (6.67)	404.4 (13.05)	384.3 (13.73)	447 (14.42)	2919 (9.73)	average monthly rainfall from 1956 to 2021 at Lockhart River Airport (Bureau of Meteorology, 2021b)
Nest water content (% w/w)	0.72	N/A	3.1	N/A	5.86	N/A	N/A	Booth D. 2020. Report to the Raine Island Recovery Project.
Estimated nest water content (% w/w)	0.65	1.22	3.23	6.08	5.79	6.69	4.46	calculated using linear regression of the rainfall and nest water content data listed here
100% male production for certain months (200 mm irrigations for producing males, 100 mm for reducing embryonic mortality)								
primary sex ratio (% male)	100^a^	100^a^	0.1	0.5	0.5	N/A	0.02	
proportion of all offspring that are male, % male	1	19	0.03	0.19	0.04	N/A	0.02	seasonal male production: 20.3%
days of irrigation needed to produce males	8	8	0	0	0	0	0	16 days of irrigation
water required to produce males (L)	218,533	4,481,240	0	0	0	0	0	4,699,773 L of water required to irrigate 1 m^2^ of sand around each nest with 200 mm rainfall equivalent
days for which nest temperatures may result in embryonic mortality	0	0	3	9	4	1	0	estimated using the number of days that the maximum air temperature was above 35°C in 2020 (Bureau of Meteorology, 2021a)
days of irrigation needed to reduce embryonic mortality	0 ^a^	0 ^a^	2	4	2	1	0	
water required to reduce embryonic mortality (L)	0 ^a^	0 ^a^	987,969	2,278,800	247,400	12,888	0	3,527,057 L of water required to irrigate 1 m^2^ of sand around each nest
total water required (L)	218,533	4,481,240	987,969	2,278,800	247,400	12,888	0	8,226,830 L of water required to produce males in October and November, and to limit embryonic mortality from December to April
estimated nest water content, % w/w	23.3	23.3	3.1	6.0	3.1	1.7	0.3	calculated using linear regression of the rainfall and nest water content data listed here
50% male production throughout the nesting season (100 mm irrigations)								
primary sex ratio, % male	50^b^	50^b^	50^b^	50^b^	50^b^	50^b^	50^b^	
proportion of all offspring that are male, % male	0.5	9.5	16.5	19	4	N/A	0.1	seasonal male production: 50%
days where irrigation was not required because of natural rainfall (maximum daily rainfall, mm)	0 (1)	1 (34.8)	6 (85.2)	3 (120.4)	2 (118.2)	5 (60.2)	1 (37.2)	estimated using the number of days that daily rainfall was above 25 mm in 2020 (Bureau of Meteorology, 2021b)
days of irrigation needed to produce males	8	8	7	7	7	7	8	irrigation occurred every 4 days except when rainfall
water required to produce males (L)	109,267	2,240,620	3,457,892	3,987,900	865,900	90,213	8067	10,759,858 L of water required to irrigate 1 m^2^ of sand around each nest
estimated nest water content (% w/w)	11.8	11.8	10.3	10.3	10.3	10.3	11.8	calculated using linear regression of the rainfall and nest water content data listed here

^a^ Irrigation occurred in these months.

^b^ Irrigation occurred in every month.

### Selecting a target incubation temperature

Nest temperatures during the TSP are typically 30—31°C from October to February (Table 2). These temperatures are 1—2°C above the pivotal temperature for northern Great Barrier Reef green turtles of ∼29.1°C (TRT 27—31°C) (I. Bell's data) (Booth et al., 2020b). To produce 100% males, temperatures during the TSP would need to be reduced by 2—3°C to ∼28°C (Booth et al., 2020b). Cooling of 2—3°C would also reduce mortality during early and late developmental stages (early‐stage green turtle embryonic thermal limit on Raine Island is ∼33°C [Booth & Dunstan, 2018]). Late‐stage embryos are generally more tolerant of heat than early‐stage embryos (Howard et al., 2014).

### Determining irrigation volume and frequency

Data to determine the relationship between the amount of cooling and irrigation volume and frequency on Raine Island are limited. Thus, we used data from Heron Island, southern Great Barrier Reef, to determine initial irrigation volumes and frequencies. Heron Island's sand composition is similar to Raine Island's (Booth et al., 2022). However, mean nest temperatures on Heron Island generally range from 28 to 31°C (Booth et al., 2013) compared with 30–31.5°C on Raine Island (Table 2); mean temperature influences how effective cooling will be. On Heron Island, a single 100‐mm application of water resulted in 1.3°C of cooling for green turtle clutches. When 100 mm of water was applied to the sand only, the sand temperature at nest depth warmed by 0.3°C (Smith et al., 2021). Thus, it seems unlikely that 100 mm of water applied to Raine Island nests would be sufficient to achieve 2°C of cooling. In comparison, 200 mm of water applied to sand on Heron Island resulted in ∼1.5°C of cooling. Thus, initially irrigating nests with 200 mm of water would provide a starting point that, with careful monitoring and adjustments, would likely provide sufficient cooling.

On Heron Island, it took 4 days for temperatures to return to preirrigation levels (Smith et al., 2021). Thus, irrigating nests every 4 days is a good starting point, although a pilot study determining the exact duration of postirrigation cooling for Raine Island is preferable. Assessment of whether this frequency leads to NWC exceeding 8–10% w/w (maximum concentration tolerated by sea turtle embryos [Patino‐Martinez et al., 2014]) and reduced hatching success is required.

Raine Island has no freshwater (Hopley, 2008). We recommend irrigating with seawater from below the thermocline because transporting freshwater from the mainland is costly, and collecting rain requires substantial infrastructure and does not supply water during the dry season (Table 3). Water from below the thermocline is cooler than surface water, increasing its cooling potential. We recommend irrigating nests from the substrate surface, potentially with agricultural sprinklers because underground systems will likely disturb nesting females (Hays & Speakman, 1993). Irrigating in the morning minimizes how much the water heats up before reaching the nest depth.

**TABLE 3:** Benefits and drawbacks of various water sources for nest irrigation^a^

Water source	Pros	Cons	Volume provided	Logistic considerations	Relative costs
Seawater	readily available no storage required minimal infrastructure	saltwater negative effects on development?	unlimited	minor, requires pumps and pipes. will depend on depth of water	low
Desalinated water powered	can produce large volumes can be powered by solar or wave action low risk of dehydration of developing embryos	requires space environmental impacts energy intensive	high. only limited by amount of water that can be processed	challenging, requires power and expensive infrastructure, plus regular maintenance	high
Desalinated water unpowered	more environmentally friendly than powered alternatives less maintenance than powered alternatives often transportable longer lasting than powered alternatives less energy intensive low risk of dehydration of developing embryos	produce limited volumes of water produces concentrated brine water may require cooling before use on nests	low	intermediate, requires some infrastructure but maintenance costs are significantly lower than powered alternatives	intermediate, much less than powered alternatives
Atmospheric extraction	low energy requirements environmentally friendly	relies on atmospheric and climatic conditions	low	intermediate, can require considerable space	intermediate, relatively low maintenance costs
Rainwater	generally, a source of cool water freshwater free capture structure could provide shade to nests	will require considerable storage dependent on rainfall does not collect the water when most needed, for example, during dry months depends on the area used to capture the rain may impact nesting birds	variable‐ depends on capture area	low	low
Transportation from the mainland	ship may be able to apply water directly to the beach via hoses freshwater	potential environmental effects of the ship will likely need multiple trips to Raine Island per nesting season may require storage potentially high greenhouse gas emissions during transport uses freshwater reserves during drought periods	high	intermediate to high, depends on storage, size of the ship, access to the island by the ship, and so on	likely high
Groundwater	less salt than seawater	brackish water, not fresh potential impacts on plants and animals on Raine Island	unknown. likely limited	intermediate to high, depends on ability to access to groundwater and impacts on the island	intermediate

^a^ The amount of water required to irrigate specific areas of Raine Island for our case study can be found in Appendix S2.

### Monitoring and adjusting the irrigation regime

Monitoring nest conditions and embryonic responses is critical to achieve desired outcomes. It is difficult to monitor PSRs in sea turtles directly (Wyneken et al., 2007). Thus, recording nest temperature on Raine Island is the most likely option for monitoring PSRs. However, temperature alone does not account for the potential direct effects of moisture on sex determination (Lolavar & Wyneken, 2017). Therefore, multiple environmental variables, particularly moisture and salinity, should be monitored throughout irrigation to ensure that embryonic mortality does not become problematic. The aim should be to maintain the targeted PSR by adjusting irrigation in response to short‐ and long‐term abiotic and biotic factors, such as air temperatures, solar radiation, and nesting density.

In the short‐term, irrigation should decrease nest temperatures and increase NWC. Hatching success should increase or remain constant, and the production of males should increase. Long‐term, the key indicators of irrigation success are altered OSRs and increased population viability. In long‐lived species that do not reach maturity for many years, juvenile sex ratios can be used as a proxy for ASRs to provide feedback on the success of irrigation.

## CURRENT KNOWLEDGE GAPS AND FUTURE RESEARCH

Few studies reported nest irrigation in sea turtles, one reported it in freshwater turtles, and, to our knowledge, none reported it in other taxa. In principle, the eggs of any oviparous species could be irrigated, but realistically, the feasibility of irrigation is determined by whether enough nests can be located and irrigated to substantially influence population characteristics and whether enough cooling can be achieved. In species that deposit eggs aboveground, any water applied may evaporate quickly, resulting in short temperature drops and no change in PSR (Yamanaka et al., 1998). Thus, irrigation may only be feasible for species that nest underground or cover their eggs (e.g., crocodiles). It is likely that irrigation will be most useful in reptiles because many nest underground and have TSD. In species with female–male–female TSD, nest temperatures will need to be monitored closely to ensure that too much cooling does not result in the production of more females. Irrigation may be useful in birds, such as the megapodes (Radley et al., 2018), and insects, including some beetles (Kirkpatrick & Sheldon, 2021). Irrigation is unlikely to be useful in mammals that are almost exclusively viviparous or amphibians that generally lay eggs directly into the water.

Studies that report embryonic responses to irrigation usually manipulate 1–2 irrigation factors, typically duration and volume, but multiple factors, including frequency, water volume, and water temperature, should be manipulated. As many of our recommended protocols as possible should be followed and reported (Table 4) to facilitate comparisons among studies, locations, and species. Irrigating larger areas may be more effective than irrigating smaller zones because the sand surrounding the nest is cooled, limiting the loss of water to the surrounding substrate. Few studies reported on seawater irrigation, and these limited the duration of irrigation to single applications (Smith et al., 2021). Further investigation of frequencies, durations, and volumes of seawater applied to nests is required.

**TABLE 4:** Recommended standard protocols when implementing, monitoring, and reporting on a nest irrigation regime

Before irrigation begins	Purpose
report initial substrate temperature, water content, and salinity	determines how much cooling is required and how much water can be applied
report substrate type and characteristics	determines how nest conditions and embryos will respond to irrigation
report both water potential (kPa) and water content (% v/v or w/w)	facilitates easier comparisons among studies and locations
report nest depth	shallower nests cool more from irrigation
report target temperature	critical when aiming to produce a particular PSR or maintain temperatures below lethal levels
report target PSR, ASR, and hatching success	dictates the amount of cooling required and thus, the target temperature
Irrigation regime details	
volume	core variables in any irrigation regime
duration	
frequency	
water source	influences water temperature and salinity
water temperature	influences the amount of cooling achieved
method of application	influences multiple factors including the volume of water needed and the cooling achieved
when during the day nests were irrigated	influences water temperature and the cooling achieved
when during development nests were irrigated	influences the response of embryos to irrigation
when during nesting season nests were irrigated	important in species that nest over a long period and when conditions change over a nesting season
During irrigation	
report changes in ambient conditions	changes in air temperatures, solar radiation and rainfall influence nest conditions and alter the response of nests to irrigation
record temperature on the sand surface	water can be heated by hot surface sand before reaching nest; measuring temperature on surface identifies how temperature of applied water changes and influences cooling
report temperature, water content, and salinity change postapplication and how much they return to previous levels before next irrigation	how long changes in incubation conditions remain postirrigation determines irrigation frequency and how much cooling can be sustained
Postirrigation	
measure embryonic and phenotypic responses	monitoring the response of embryos key to determining the effectiveness of irrigation regime
measure PSR directly	if possible measure PSR directly rather than using temperature as a proxy to account for variation among clutches and other unknown variation
report how long it takes temperature, water content, and salinity to return to preirrigation levels	determines whether irrigation duration can be shortened or needs to be longer
report nest conditions and phenotypic responses of control nests	provides contrast to assess how effective irrigation was; accounts for variation in ambient conditions more than using initial conditions in irrigated nests

Abbreviations: ASR, adult sex ration; PSR, primary sex ration.

Nest irrigation can cool developing embryos in response to climate change. However, multiple factors, including ambient conditions, substrate, and species characteristics, can influence the cooling achieved. Thus, generalizing among studies is difficult. Irrigation volume showed the largest effect on cooling, but larger volumes increased the risk of inundation. The major drawback of irrigation is the requirement for a water source, particularly in remote and dry locations, that can supply enough water to maintain enough cooling. Ambient conditions, nesting density, and stage of embryonic development variations within and among nesting seasons are critical to monitor because they alter how embryos respond to irrigation.

## Supporting information

Table S1: How likely various factors are to influence the response of nests and embryos to irrigation. For example, how the level of shade influences how nest salinity responds to irrigation. We do not include information on rainfall events or tidal inundation in this table.
Table S2: Potential target sections for irrigation and the volume of water, capture area, and storage required.
