# Supplementary material for: Mitigating the effects of climate change on the nests of sea turtles with artificial irrigation
Source: Conserv Biol. 2023 Jan 22;37(1):e14044. doi: 10.1111/cobi.14044 (PMC10108123; doi:10.1111/cobi.14044)
Supplement: Supplementary file 1 — Table S1: How likely various factors are to influence the response of nests and embryos to irrigation. For example, how the level of shade influences how nest salinity responds to irrigation. We do not include information on rainfall events or tidal inundation in this table. Table S2: Potential target sections for irrigation and the volume of water, capture area, and storage required. [file COBI-37-0-s001.docx]

Table S1: How likely various factors are to influence the response of nests and embryos to irrigation. For example, how the level of shade influences how nest salinity responds to irrigation. We do not include information on rainfall events or tidal inundation in this table.

|  | **Nest temperature** | **Duration of temperature change** | | **Nest moisture** | **Nest salinity** | **Incubation period** | **Hatching success** | **Primary sex ratio** | **Hatchling morphology** | **Hatchling locomotor performance** |
| --- | --- | --- | --- | --- | --- | --- | --- | --- | --- | --- |
|  |  | **From the start of irrigation** | **Post-irrigation only** |  |  |  |  |  |  |  |
| **Nest depth** | Usually | Unknown | | Usually | Usually | Likely | | | | |
| **Shade** | Usually | Unknown | | Usually | Usually | Likely | | | | |
| **Method of application** | Unknown- studies on irrigation exclusively report surface irrigation. | | | | | | | | | |
| **Water source** | No | No | | No | Usually | Unlikely | Likely | Unlikely | Likely | Likely |
| **Water temperature** | Usually | Unlikely | | No | No | Likely | | | | |
| **Time of day** | Likely | Unlikely | | Unlikely | Unlikely | Likely | | | | |
| **Time during incubation** | Possible ^A^ | Unlikely | | Unlikely | Unlikely | Likely | | | | |
| **Time during the nesting season** | Likely | Unlikely | | Likely | Likely | Likely | | | | |
| **Volume** | Usually | Unlikely | Unlikely | Usually | Possible ^B^ | Likely | | | | |
| **Frequency** | Usually | Usually | Unlikely | Usually | Possible ^B^ | Likely | | | | |
| **Duration** | Usually | Usually | Unlikely | Usually | Possible ^B^ | Likely | | | | |

^A^ Depends on the amount of metabolic heating

^B^ Depends on the salinity of the water source and/or the amount of salt already present in the nest

Table S2: Potential target sections for irrigation and the volume of water, capture area and storage required.

| Irrigating: | Dimensions | Total area (m^2^) | Water required for 100 mm rainfall equivalent of irrigation (L) | Capture area required ^D^ (m^2^) | Size of a storage unit (m) ^E^ |
| --- | --- | --- | --- | --- | --- |
| All non-vegetated areas | 40% of Raine Island ^A^ | ~108,000 | 10,800,000 | ~27,000 | Diameter: ~30  Height: ~15 |
| Re-profiled areas (2014 & 2017) | 1: 150 x 100m  2: 250 x 100m ^B^ | 40,000 | 4,000,000 | ~10,000 | Diameter: ~22  Height: ~11.4 |
| Re-profiled areas (2014, 2017 & 2019) | Sections 1 & 2 plus:  3. 160 x 65m  4: 160 x 40m ^C^ | 56,800 | 5,680,000 | ~14,000 | Diameter: ~22  Height: ~14.5 |

^A^ Hopley (2008)

^B^ Dunstan and Roberston (2019)

^C^ Dunstan et al. (2020)

^D^ Area required to capture enough water for a single 100mm application of water based on the average rainfall for January 15 to the end of February (404mm; Dunstan & Roberston 2018). This period is likely to have the highest number of clutches incubating, lowest male production and coincides with the warmest and wettest part of the nesting season (Limpus et al. 2003, Booth et al. 2020b)

^E^ Dimensions of a cylindrical tank large enough to hold enough water for a single 100mm application of water
